# Management of long-term and reversible hysteroscopic sterilization: a novel device with nickel-titanium shape memory alloy

**DOI:** 10.1186/1477-7827-12-61

**Published:** 2014-07-07

**Authors:** Bin Xu, Ke-an Zhu, Dabao Xu, Aixingzi Aili

**Affiliations:** 1 Department of Gynecology, Third Xiangya Hospital of Central South University, 138 Tongzipo Rd, Changsha City, Hunan Province 410013, China; 2 Department of Gynecology, Shanghai East Hospital of Tongji University, 150 Jimo Rd, Shanghai 200120, China

**Keywords:** Nickel-titanium alloy, Shape memory, Hysteroscopic sterilization, Contraception, Reversibility, In vitro fertilization

## Abstract

**Background:**

Female sterilization is the second most commonly used method of contraception in the United States. Female sterilization can now be performed through laparoscopic, abdominal, or hysteroscopic approaches. The hysteroscopic sterilization may be a safer option than sterilization through laparoscopy or laparotomy because it avoids invading the abdominal cavity and undergoing general anaesthesia. Hysteroscopic sterilization mainly includes chemical agents and mechanical devices. Common issues related to the toxicity of the chemical agents used have raised concerns regarding this kind of contraception. The difficulty of the transcervical insertion of such mechanical devices into the fallopian tubes has increased the high incidence of device displacement or dislodgment. At present, Essure^®^ is the only commercially available hysteroscopic sterilization device being used clinically. The system is irreversible and is not effective immediately.

**Presentation of the hypothesis:**

Our new hysteroscopic sterility system consists of nickel-titanium (NiTi) shape memory alloy and a waterproof membrane. The NiTi alloy is covered with two coatings to avoid toxic Ni release and to prevent stimulation of epithelial tissue growth around the oviducts. Because of the shape memory effect of the NiTi alloy, the device works like an umbrella: it stays collapsed at low temperature before placement and opens by the force of shape memory activated by the body temperature after it is inserted hysteroscopically into the interstitial tubal lumen. The rim of the open device will incise into interstitial myometrium during the process of unfolding. Once the device is fixed, it blocks the tube completely. When the patient no longer wishes for sterilization, the device can be closed by perfusing liquid with low temperature into the uterine cavity, followed by prospective hysteroscopic removal. After the device removal, the fallopian tube will revert to its physiological functions.

**Testing the hypothesis:**

Currently, experimental and clinical studies are needed to attest the safety, efficiency and reversibility of the novel sterilization device.

**Implications of the hypothesis:**

If our hypothesis is confirmed, appropriate and reversible contraceptive can be achieved with the device we have designed, which may have significant repercussions for numerous women worldwide.

## Background

Hysteroscopic sterilization is a safer option than sterilization through laparoscopy or laparotomy because it avoids abdominal cavity invasion and the use of general anaesthesia. Moreover, this procedure can be performed in an outpatient setting by an incision-free route, with a shorter recovery period, and at a lower cost [1-4]. Scholars around the world devote themselves to the research of new technologies for female sterilization, including methods that involve tubal occlusion, mainly by using chemical agents combined with a sclerosing agent [5], cyanoacrylate [6], n-butyl-2-cyanoacrylate adhesive [7], and polidocanol [8]. Other mechanical devices such as in situ-gelling polymeric device [9], implant with two sets of six slots [10], or intestine submucosal device [11] are also under investigation. The main concerns are related to the toxicity of the chemical agents on the tubal epithelium, peritoneum, and pelvic organs. Additionally, the transcervical insertion of the mechanical device into fallopian tube may be technically challenging, resulting in a high incidence of device displacement or dislodgment [12]. The methods mentioned above are not currently in use in a clinical setting.

Gradually, with the development of material science, shape memory materials (SMMs), including shape memory alloys (SMAs) and shape memory polymers (SMPs), have attracted a lot of attention because of their unique characteristics and super elasticity. SMMs have the capability of “memorizing” and recovering their permanent shape (SP) from an original temporary shape when subjected to an appropriate temperature or other particular stimuli. Therefore, a bulky device could potentially be implanted in a relatively small space in a compressed temporary shape through less invasive surgery. Once in place, it can be expanded to its previously defined size or shape to fit the space as required [13-16]. The application of SMMs for the sterilization purposes, two studies including a SMP device [12] and an oviduct plugs with nickel-titanium (NiTi) shape memory alloy and silicone rubber [17] are under study in animal experiments in China. Additionally, Essure^®^ (Conceptus Incorporated, Mountain View, CA, USA) is a most promising transcervical sterilization device, and its body is made of NiTi shape memory alloy [18]. This system was approved by the US Food and Drug Administration (FDA) in 2002. Currently, it is the only commercially available hysteroscopic sterilization device used in clinical practice, since the Adiana^®^ Permanent Contraception System (Hologic, Inc., Bedford, MA, USA), approved by the FDA in 2009, was removed from the US market by the manufacturer in 2012 [19,20]. Both Essure^®^ and Adiana^®^ contraception systems were proven efficacious and safe by relatively short-term clinical trial data. Very few pregnancies occurred among women with confirmed bilateral tubal occlusion achieved with both hysteroscopic methods. However, long-term data beyond 7 years is unavailable. According to the FDA directions and product labelling, bilateral tubal occlusion must be confirmed 3 months after device implantation mainly by performing hysterosalpingogram (HSG). At this time, the fallopian tubes undergo fibrosis, which leads to tubal occlusion and causes the contraceptive effect. Thus, patients must use alternative contraception for at least 3 months post procedure. Even at that time, if bilateral tubal occlusion is not confirmed, the patient should continue alternative contraception for 3 additional months. Pregnancies that occurred while either one of these devices was in place were attributed to lack of follow-up, improper use of alternative contraception, failure of alternative contraception, or misinterpretation of HSG results [19-21].

Aside from the lack of immediate effect, another limitation is that both systems approved by FDA are irreversible. In fact, in the United States, the overall cumulative probability of expressing regret within 14 years after tubal sterilization was 20.3% for women aged 30 years or younger and 5.9% for women over the age of 30 years at the time of sterilization. However, after permanent hysteroscopic sterilization, the opportunity to undergo sterilization reversal seems unlikely. Some requests for sterilization reversal were followed by in vitro fertilization (IVF) procedures. However, the impact of the Essure^®^ coil on IVF outcomes and pregnancy course is uncertain [22,23]. Therefore, we thought of developing a reversible and immediately effective hysteroscopic sterilization method that is safe and effective. In the present article, we describe a micro-barrier contraception method by using shape memory alloy that can be easily inserted in the interstitial fallopian tube in an outpatient setting. Our proposed method may result in a low-cost, non-hormonal, long-term, reversible, and discreet method of contraception for women.

## The presentation of hypothesis

The manufacture of the hysteroscopic sterilization device.

The device consists of an umbrella supporter made of NiTi alloy and an umbrella fabric made of a waterproof polyurethane-like membrane. Our device is 8 mm in length and 1.5 mm in diameter when closed. Once opened, the diameter of the umbrella fabric expands to 6.5 mm. One end (head) of the device is covered with a semi-soft cap made of a biodegradable material. At the other end (tail), there is a small fibre intended to facilitate removal. The NiTi alloy is covered with two coatings. The first coating is silicon dioxide (SiO_2_) applied using the sol–gel method to avoid toxic Ni release. The second coating is a macromolecular substance with high biological stability to prevent stimulation of epithelial tissue of the oviduct growth. The candidates for the second coating include poly (dimethylacrylamide) (PDMAA) and poly (2-ethyloxazoline) (PEtOx), which represent anti-adhesive properties and can be applied as coatings by a photochemical grafting method to reduce the unspecific extracellular matrix-mediated adhesion of fibroblasts to implant surfaces [24].

The device can change its size according to the temperature.

**Figure 1:**
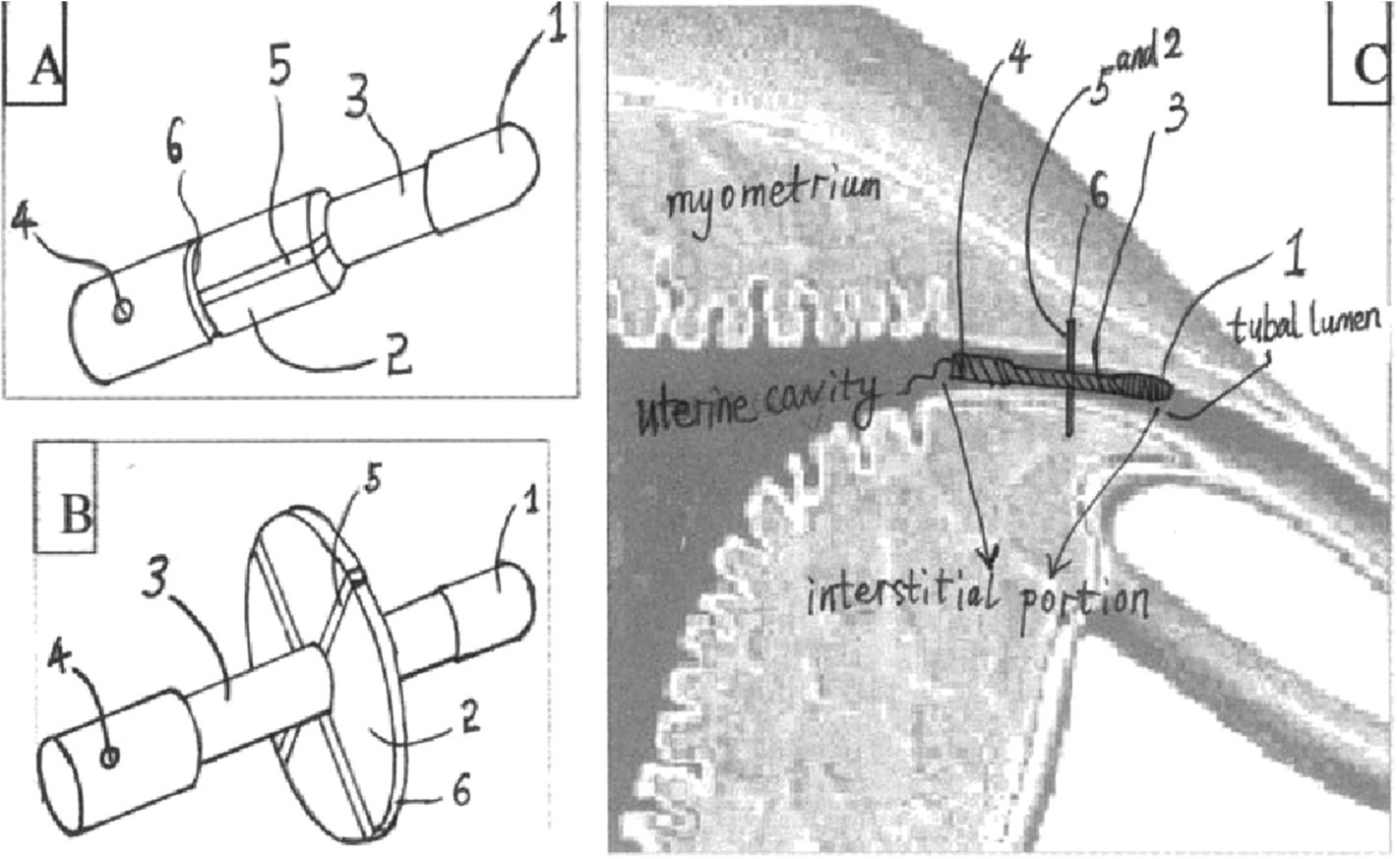
**Illustration of the structure and working principle of the device. (A)** The device before insertion into the tubal interstitial portion, like a folded umbrella which minimizes its size in diameter and subsequently facilitates the insertion of the device; **(B)** The device after the insertion into the interstitial portion, like an unfolded umbrella; **(C)** Coronal section view, the device is placed at the interstitial portion of the tube, The device is anchored in the right place and the passage through the tube is completely blocked. 1. A semi-soft hat; 2. A waterproof membrane; 3. Guild rod; 4. A small hole; 5. The supporter of the shape memory umbrella; 6. A thin circular rim to cut into the interstitial myometrium for fixation.

NiTi alloy is a SMA, which “remembers” its original and forged shape at a specified temperature. Basically, SMA presents two well-defined phases: austenite and martensite. The martensite phase consists of a SMA only at low temperature range. Martensite is soft and can be easily deformed by stress. When the temperature rises to austenite activation temperature (*As*, the lowest temperature at which the SMA starts to change its shape), the SMA recovers its shape toward the original and forged shape gradually and completely when the temperature reaches austenite final temperature (*Af*). Then, when temperature decreases, austenite starts to change its shape to martensite at martensite start temperature (Ms) and completes the transformation at martensite final temperature (Mf) [14,25]. The device is like an umbrella that stays collapsed before insertion; it is activated by body temperature and subsequently opened by the force of shape memory (see Figure 1). The umbrella fabric is supported by several axes joined with a thin circular ring and covered with the waterproof polyurethane-like membrane. The opened device anchors in the interstitial fallopian tube as a barrier and prevents the sperm cells from coming in contact with the ova.

The device can be placed hysteroscopically.

According to our hypothesis, when the device is inserted via hysteroscopy, the temperature of the running distension fluid into the uterine cavity is low; thus, the device maintains its collapsed shape. After it is inserted into the interstitial tubal lumen, the device temperature rises to body temperature; consequently, the device “remembers” and returns to its originally forged shape. The *rim* of the open device will incise into the interstitial myometrium during the process of unfolding. The rim is very thin, which facilitates the cut. Once the incision process is finished, the device is fixed to the interstitial myometrium. The open device will obstruct the tube completely.

The device is a reversible sterilization method.

The device anchors in a very narrow part of the tube and little tissue is damaged by the thin rim. Because the device is coated with a highly stable macromolecular substance, it is biologically inactive and cannot induce tissue growth or encapsulation. When the patient wishes to reverse sterilization, a liquid at low temperature is perfused into the uterine cavity. Then device tail can be used for its removal under hysteroscopy. Owing to litter damage caused by the thin rim (diameter: 0.05-0.1 mm), the scars in the fallopian tube will be very small. The fallopian tube will revert to its function after the device is removed.

Therefore, the design of the safe, reversible, and immediately effective sterilization device was achieved. We obtained a national patent in China for the invention of the sterilization device described herein. The application number of the patent is ZL200810030905 [26] (see Additional file 1: supplemental document S1 and Additional file 2: supplemental document S2).

## Testing the hypothesis

The core for testing our hypothesis is to successfully manufacture the barrier contraceptive device by using shape memory material and to test the device activation and modification under different temperatures simulating a normal range of body temperatures at our material laboratory. The testing process involved the following steps.

Step 1, to manufacture the device parts, including the following:

(1) Manufacture the NiTi alloy adjust the NiTi alloy *As* to 10 degrees centigrade and *Af* to 35 degrees centigrade by changing the mixture proportions of Ni and Ti and by applying a thermal treatment technique in the laboratory;

(2) Manufacture the NiTi alloy supporter;

(3) Cover with both coatings and test the efficiency and biocompatibility of the coatings;

(4) Manufacture the waterproof membrane made of poly(dimethylacrylamide) (PDMAA) or poly(2-ethyloxazoline) (PEtOx);

(5) Assemble the device supporter made of NiTi alloy and the waterproof membrane.

Step 2, to manufacture 6 devices and test the effect of the transformation from the closed shape to the opened shape under simulated temperatures (under water) in the material laboratory.

Step 3, to test the placement of the device and the effect of the transformation hysteroscopically under simulated temperatures in a surgically excised uterus: placing the device into the interstitial portion in a surgically excised uterus (with interstitial portions) by using the hysteroscopic catheterization technique.

Step 4, to conduct prospective, observational animal experiments and then clinical studies to assess device safety, efficacy and reversibility, that is, we will continue to test the device and collect data on biocompatibility, successful placement rate, obstruction rate, the contraceptive effect and reversal of contraceptive effect, and any complications (such as nickel allergies, infection, pain, and device displacement) in animal experiments using goats and then a preliminary clinical trial with a small sample size (about 10 to 15 patients: age ≥ 20 years, gravity ≥ 1, without infertility). Necessarily, we will adjust and improve the design of the device. If the preliminary clinical trial presents the device is safe and effective, a large scale clinical trial (at least 50 to 100 patients) will be conducted to evaluate the device comprehensively.

## Implications of the hypothesis

It is predicted that the total world population will reach 8.9 billion in 2050 and more than 26 billion couples will need contraceptives in the next half century. Although steady progress in hormonal and non-hormonal modern contraceptive research has been achieved over the past 50 years, the contraceptives available today have all kinds of adverse effects and are not suitable for all users. A need to expand contraceptive methods still exists [27,28]. Our efforts to develop a novel contraceptive device with special characteristics make great sense. Two of the largest advantages of this contraception method are reversibility and immediate effectiveness. Essure^®^, the most promising intratubal device available, consists of a stainless steel inner coil, an outer coil of nitinol, and polyethylene terephthalate (PET) fibres. After insertion into the interstitial portion of each fallopian tube by hysteroscopic guidance, the outer coil expanded and anchored the device to the fallopian tube. Over the next few weeks, fibrotic tissue growth around the device by PET stimulating results in complete tubal occlusion [29,30]. At last, tubal occlusion and proper positioning must be confirmed at 3 months postprocedure by performing a HSG, or a pelvic radiograph for both sterilization systems [30]. Owing to the irreversible damage of the tube, the sterilization methods are permanent. If patients wish to conceive again, they must turn to IVF. Obviously, our reversible device is more practical and allows patients to change their minds concerning sterilization. Our device is the less invasive modality. Our device, with a uniquely designed shape, is 8 mm in length, equivalent to the interstitial portion of fallopian tube. There is no pressure from the oviduct peristalsis and the device is relatively unlikely to be excluded by this force. The difficulty of placement and possibility of perforation are lower. However, Essure^®^, for example, is 4 cm long and anchors to a 3-cm segment of the tube. Bilateral tube placement would fail if there is adhesion in the isthmus or ampulla portions of the fallopian tube. In addition, several studies reported the use of Essure^®^ devices in proximal tubal occlusion in subfertile women with hydrosalpinges before IVF [31] and receiving IVF procedures with the presence of the Essure^®^[32]. Although, researchers favoured the outcome, the sample size was still very small. Further, there is concern that intrauterine coils of Essure^®^ could interfere with subsequent IVF procedures [29]. Therefore, a safe and effective hysteroscopic sterilization method is needed urgently.

In theory, the development consists of attractive advantages such as the long-term, easy-placement, reversible and immediately effective. If it is really effective, it may have significant repercussions for numerous women worldwide. However, the device obstructs the fallopian tube mechanically by a waterproof polyurethane-like membrane rather than destroys the cavity of tube and makes a permanent obstruction by the formation of fibrotic tissue as Essure^®^. Theoretically, our device would have a lower contraceptive success rate. Therefore, the corrosion resistance, stability and tenacity of the waterproof membrane need further research. In addition, whether the scars in the fallopian tube caused by the device placement could lead to impaired tubal motility and, thereby, impact on fertility negatively after removal of the devices also is an important part of our study in the future. For these reasons, repeated animal experiments and subsequent clinical studies are now needed to attest the safety and efficiency of the novel contraceptive device and improve it continuously.

## Competing interests

The authors declare that they have no competing interests.

## Authors’ contributions

XB wrote the manuscript. XDB and ALA conceived the study. ZKA, XDB and ALA participated in its design and helped to draft the manuscript. All authors read and approved the final manuscript.

## Supplementary Material

Additional file 1 **Supplemental document S1.** The original copy of patent approval (in Chinese). Click here for file

Additional file 2 **Supplemental document S2.** The certification copy of patent approval (in English). Click here for file
