# Supplementary material for: Management of long-term and reversible hysteroscopic sterilization: a novel device with nickel-titanium shape memory alloy
Source: Reprod Biol Endocrinol. 2014 Jul 7;12:61. doi: 10.1186/1477-7827-12-61 (PMC4105153; doi:10.1186/1477-7827-12-61)
Supplement: Additional file 1 — Supplemental document S1. The original copy of patent approval (in Chinese). [file 1477-7827-12-61-S1.pdf]

(19)中华人民共和国国家知识产权局

(12)发明专利

(10)授权公告号 CN 101249032 B
(45)授权公告日 2010.04.21

(21)申请号 200810030905.8
(22)申请日 2008.03.25
(73)专利权人 朱东波
地址 410083 湖南省长沙市岳麓区中南大学本部科技处
专利权人 徐大宝
刘芳
(72)发明人 徐大宝
(51)Int.Cl.
*A61F 6/22*(2006.01)
(56)对比文件
CN 201168109 Y,2008.12.24, 权利要求 1-5.
US 3645258 ,1972.02.29, 说明书第 1 栏第 35-40 行,附图 4-5.
JP 昭 57-75648 A,1982.05.12, 全文 .
CN 1919161 A,2007.02.28, 权利要求 1,4、说明书具体实施方式的第 2-3 段、附图 1.
CN 1596846 A,2005.03.23, 全文 .
CN 1334068 A,2002.02.06, 全文 .
US 4004582 ,1977.01.25, 全文 .
US 4094313 ,1978.06.13, 全文 .
CN 2848183 Y,2006.12.20, 权利要求 1,3,5,6、说明书第 1 页第 4-5 段、附图 1、2.
审查员 胡亚婷

权利要求书 1 页 说明书 3 页 附图 1 页

(54)发明名称

绝育栓

(57)摘要

本发明公开了一种绝育栓。包括导引杆、至少一个支架、以及薄膜,支架环绕连接在导引杆上,薄膜粘附在支架上,导引杆和粘附在支架上的薄膜横截面为圆面,导引杆的长度为 4mm 至 15mm,直径为 0.2mm 至 1.8mm,所述支架呈放射状,每根放射状支架的长度为 1mm 至 5mm,厚度为 0.1mm 至 0.8mm。由于导引杆可方便地置入输卵管的间质部,放射状支架的顶端可嵌入肌层,不会使绝育栓移动和脱落,粘附在支架上的薄膜可有效地阻止精子进入输卵管,达到绝育的效果并可达到很高的绝育成功率。由于放射状支架较之截面为实体平面的突起容易变形,因此本发明具有变形容易,对人体副作用小,以及制备简单,成本低的有益效果。

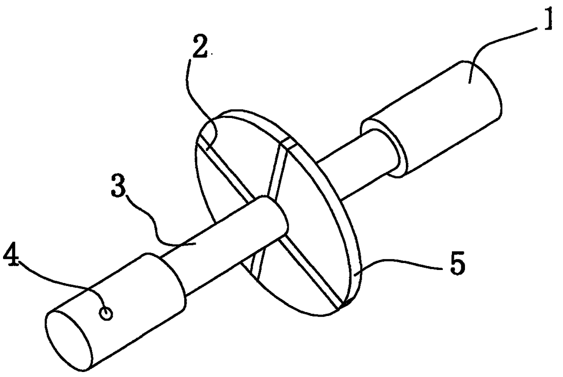


CN 101249032 B

1. 一种绝育栓,包括导引杆、至少一个支架、以及薄膜,支架环绕连接在导引杆上,薄膜粘附在支架上,导引杆和粘附在支架上的薄膜横截面为圆面,导引杆的长度为 4mm 至 15mm,直径为 0.2mm 至 1.8mm,其特征在于,所述支架呈放射状,每根放射状支架的长度为 1mm 至 5mm,厚度为 0.1mm 至 0.8mm。

2. 根据权利要求 1 的绝育栓,其特征在于,在导引杆表面上至少靠近放射状支架的一边有环绕导引杆的沟槽,沟槽的长度为 1mm 至 5mm,沟槽部分所在的导引杆的直径为 0.1mm 至 1.7mm。

3. 根据权利要求 1 或者 2 的绝育栓,其特征在于,在导引杆上有一孔。

4. 根据权利要求 1 或者 2 的绝育栓,其特征在于,所述绝育栓由 TiNi 合金制成,其中 Ni 的含量为 56%～58%,余量为 Ti。

# 绝育栓

## 技术领域

[0001] 本发明涉及一种绝育栓，特别是置于输卵管间质部的绝育栓。

## 背景技术

[0002] 经宫腔镜输卵管绝育术因为不需在人体腹部开刀，避免了腹部切口和内脏器官的损伤，具有广阔的应用前景。中国专利 200520051371.9 公开了一种由形状记忆合金制成的绝育栓，其包括导引杆和环绕连接在导引杆上的至少一个突起。它的优点在于绝育成功率高，放置简单，且对人体副作用小。但是由于导引杆上的突起截面为实体平面，沿导引杆轴向不易变形，因此对于形状记忆合金材质及其加工的要求很高，因此成本居高不下。

## 发明内容

[0003] 本发明的目的是提供一种变形容易，制备简单，成本低的绝育栓。

[0004] 为了达到上述目的，本发明的绝育栓包括导引杆、至少一个支架、以及薄膜，支架环绕连接在导引杆上，薄膜粘附在支架上，导引杆和粘附在支架上的薄膜横截面为圆面，导引杆的长度为 4mm 至 15mm，直径为 0.2mm 至 1.8mm，所述支架呈放射状，每根放射状支架的长度为 1mm 至 5mm，厚度为 0.1mm 至 0.8mm。

[0005] 在导引杆表面上至少靠近放射状支架的一边有环绕导引杆的沟槽，沟槽的长度为 1mm 至 5mm，沟槽部分所在的导引杆的直径为 0.1mm 至 1.7mm。

[0006] 在导引杆上有一孔，便于放入和取出绝育栓。

[0007] 所述绝育栓由 TiNi 合金制成，其中 Ni 的含量为 56%～ 58%，余量为 Ti。

[0008] 本发明的绝育栓导引杆可方便地置入输卵管的间质部，放射状支架的顶端可嵌入肌层，不会使绝育栓移动和脱落，粘附在支架上的薄膜可有效地阻止精子进入输卵管，达到绝育的效果并可达到很高的绝育的成功率。由于放射状支架较之截面为实体平面的突起容易变形，因此本发明具有变形容易的有益效果。同时，放射状支架的顶端与输卵管的接触面很小，不会对其造成较大损伤，因而对人体的副作用很小。另外本发明还有制备简单，成本低的优点。

## 附图说明

[0009] 图 1 是本发明绝育栓的一个具体实施方式的立体图。

## 具体实施方式

[0010] 实施例一：如图 1 所示，本发明绝育栓由 TiNi 合金制成，其中 Ni 的重量百分含量为 56%，余量为 Ti。绝育栓包括导引杆 1 和环绕连接在导引杆 1 上的四个放射状支架 2，支架 2 上粘附有薄膜 5。导引杆 1 和粘附在支架 2 上的薄膜 5 横截面为圆面。导引杆 1 的长度为 4mm，直径为 0.2mm，每根放射状支架 2 的长度为 1mm，厚度为 0.1mm。在导引杆 1 表面上至少靠近支架 2 的一边有环绕导引杆 1 的沟槽 3。沟槽 3 的长度为 1mm，沟槽 3 部分所在

的导引杆 1 的直径为 0.1mm。薄膜 5 粘附在支架 2 上，其尺寸与放射状支架 2 相适应。支架 2 可在软化温度 10℃下折入沟槽中，在将绝育栓置入人体输卵管的间质部后，在接近人体体温的形状记忆温度下，支架 2 可恢复原有的形状，其顶端嵌入输卵管间质层的肌层，不会使绝育栓移动和脱落。同时又由于粘附在支架上的薄膜与输卵管的内壁之间无间隙，有效堵塞了输卵管，这样可达到很高的绝育的成功率。在导引杆上有－孔 4，可以用来栓线，方便了绝育栓的放置和取出。

[0011] 实施例二：本发明绝育栓由 TiNi 合金制成，其中 Ni 的重量百分含量为 56.5％，余量为 Ti。绝育栓包括导引杆 1 和环绕连接在导引杆 1 上的六个放射状支架 2，支架 2 上粘附有薄膜 5。导引杆 1 和粘附在支架 2 上的薄膜 5 横截面为圆面。导引杆 1 的长度为 6mm，直径为 0.5mm，每根放射状支架 2 的长度为 2mm，厚度为 0.3mm。在导引杆 1 表面上至少靠近支架 2 的一边有环绕导引杆 1 的沟槽 3。沟槽 3 的长度为 2mm，沟槽 3 部分所在的导引杆 1 的直径为 0.2mm。薄膜 5 粘附在支架 2 上，其尺寸与放射状支架 2 相适应。支架 2 可在软化温度 10℃下折入沟槽中，在将绝育栓置入人体输卵管的间质部后，在接近人体体温的形状记忆温度下，支架 2 可恢复原有的形状，其顶端嵌入输卵管间质层的肌层，不会使绝育栓移动和脱落。同时又由于粘附在支架上的薄膜与输卵管的内壁之间无间隙，有效堵塞了输卵管，这样可达到很高的绝育的成功率。在导引杆上有一孔 4，可以用来栓线，方便了绝育栓的放置和取出。

[0012] 实施例三：本发明绝育栓由 TiNi 合金制成，其中 Ni 的重量百分含量为 57.3％，余量为 Ti。绝育栓包括导引杆 1 和环绕连接在导引杆 1 上的六个放射状支架 2，支架 2 上粘附有薄膜 5。导引杆 1 和粘附在支架 2 上的薄膜 5 横截面为圆面。导引杆 1 的长度为 10mm，直径为 1.8mm，每根放射状支架 2 的长度为 3mm，厚度为 0.5mm。在导引杆 1 表面上至少靠近支架 2 的一边有环绕导引杆 1 的沟槽 3。沟槽 3 的长度为 3mm，沟槽 3 部分所在的导引杆 1 的直径为 1.3mm。薄膜 5 粘附在支架 2 上，其尺寸与放射状支架 2 相适应。支架 2 可在软化温度 10℃下折入沟槽中，在将绝育栓置入人体输卵管的间质部后，在接近人体体温的形状记忆温度下，支架 2 可恢复原有的形状，其顶端嵌入输卵管间质层的肌层，不会使绝育栓移动和脱落。同时又由于粘附在支架上的薄膜与输卵管的内壁之间无间隙，有效堵塞了输卵管，这样可达到很高的绝育的成功率。在导引杆上有一孔 4，可以用来栓线，方便了绝育栓的放置和取出。

[0013] 实施例四：本发明绝育栓由 TiNi 合金制成，其中 Ni 的重量百分含量为 57.6％，余量为 Ti。绝育栓包括导引杆 1 和环绕连接在导引杆 1 上的六个放射状支架 2，支架 2 上粘附有薄膜 5。导引杆 1 和粘附在支架 2 上的薄膜 5 横截面为圆面。导引杆 1 的长度为 15mm，直径为 1.4mm，每根放射状支架 2 的长度为 5mm，厚度为 0.8mm。在导引杆 1 表面上至少靠近支架 2 的一边有环绕导引杆 1 的沟槽 3。沟槽 3 的长度为 5mm，沟槽 3 部分所在的导引杆 1 的直径为 0.6mm。薄膜 5 粘附在支架 2 上，其尺寸与放射状支架 2 相适应。支架 2 可在软化温度 10℃下折入沟槽中，在将绝育栓置入人体输卵管的间质部后，在接近人体体温的形状记忆温度下，支架 2 可恢复原有的形状，其顶端嵌入输卵管间质层的肌层，不会使绝育栓移动和脱落。同时又由于粘附在支架上的薄膜与输卵管的内壁之间无间隙，有效堵塞了输卵管，这样可达到很高的绝育的成功率。在导引杆上有一孔 4，可以用来栓线，方便了绝育栓的放置和取出。

[0014] 实施例五：本发明绝育栓由 TiNi 合金制成，其中 Ni 的重量百分含量为 58％，余量为 Ti。绝育栓包括导引杆 1 和环绕连接在导引杆 1 上的六个放射状支架 2，支架 2 上粘附有薄膜 5。导引杆 1 和粘附在支架 2 上的薄膜 5 横截面为圆面。导引杆 1 的长度为 8mm，直径为 1.8mm，每根放射状支架的长度为 2.5mm，厚度为 0.1mm。在导引杆 1 表面上至少靠近支架 2 的一边有环绕导引杆 1 的沟槽 3。沟槽 3 的长度为 2.5mm，沟槽 3 部分所在的导引杆 1 的直径为 1.7mm。薄膜 5 粘附在支架 2 上，其尺寸与放射状支架 2 相适应。支架 2 可在软化温度 10℃下折入沟槽中，在将绝育栓置入人体输卵管的间质部后，在接近人体体温的形状记忆温度下，支架 2 可恢复原有的形状，其顶端嵌入输卵管间质层的肌层，不会使绝育栓移动和脱落。同时又由于粘附在支架上的薄膜与输卵管的内壁之间无间隙，有效堵塞了输卵管，这样可达到很高的绝育的成功率。在导引杆上有一孔 4，可以用来栓线，方便了绝育栓的放置和取出。

[0015] 由于本发明由具有形状记忆功能的材料 TINi 合金制成，可在低于软化温度下方便地将其从人体输卵管的间质部取出，而使人恢复受孕功能。该绝育栓还可反复使用。

[0016] 本发明的绝育栓的放射状支架的截面形状可为条形、齿形或者扇形。粘附在支架上的薄膜可为尼龙材料等人体适合的材料，只要是满足本发明技术方案所述内容，都在本专利保护范围之内。

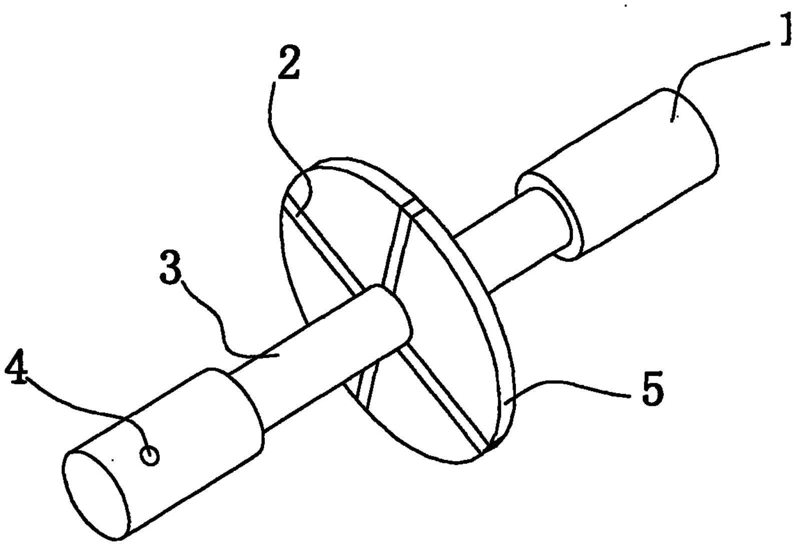


图 1
