# Supplementary material for: Management of long-term and reversible hysteroscopic sterilization: a novel device with nickel-titanium shape memory alloy
Source: Reprod Biol Endocrinol. 2014 Jul 7;12:61. doi: 10.1186/1477-7827-12-61 (PMC4105153; doi:10.1186/1477-7827-12-61)
Supplement: Additional file 2 — Supplemental document S2. The certification copy of patent approval (in English). [file 1477-7827-12-61-S2.doc]

Invention Title -- Sterilization bolt

| Application No. | CN200810030905 |
| --- | --- |
| Application Date | 2008.03.25 |
| Publication No. | CN101249032B |
| Publication Date | 2010.04.21 |
| IPC Classification No. | A61F6/22 |
| Applicant/ Assignee | ZHU DONGBO; DABAO XU; LIU FANG; |
| Inventor | DABAO XU; |
| Priority No. | CN200810030905 |
| Priority Date | 2008.03.25 |

**Abstract**

The invention discloses a contraceptive plug, which comprises a guide rod, at least one radial-pattern supporter windingly connected with the guide rod, and a film attached to the supporter. The guide rod can be easily placed in the interstitial portion of fallopian tube, the top end of the radial-pattern supporter can be embedded in uterine muscles to prevent the contraceptive plug from moving and falling, and the film attached to the supporter can efficiently prevent sperms entering the fallopian tube, so as to achieve contraceptive effect and achieve high success rate of contraception. Because the radial-pattern supporter is deformable compared with bumps with solid planar cross section, therefore, the inventive contraceptive plug has good flexibility.

**Graphs**

窗体顶端


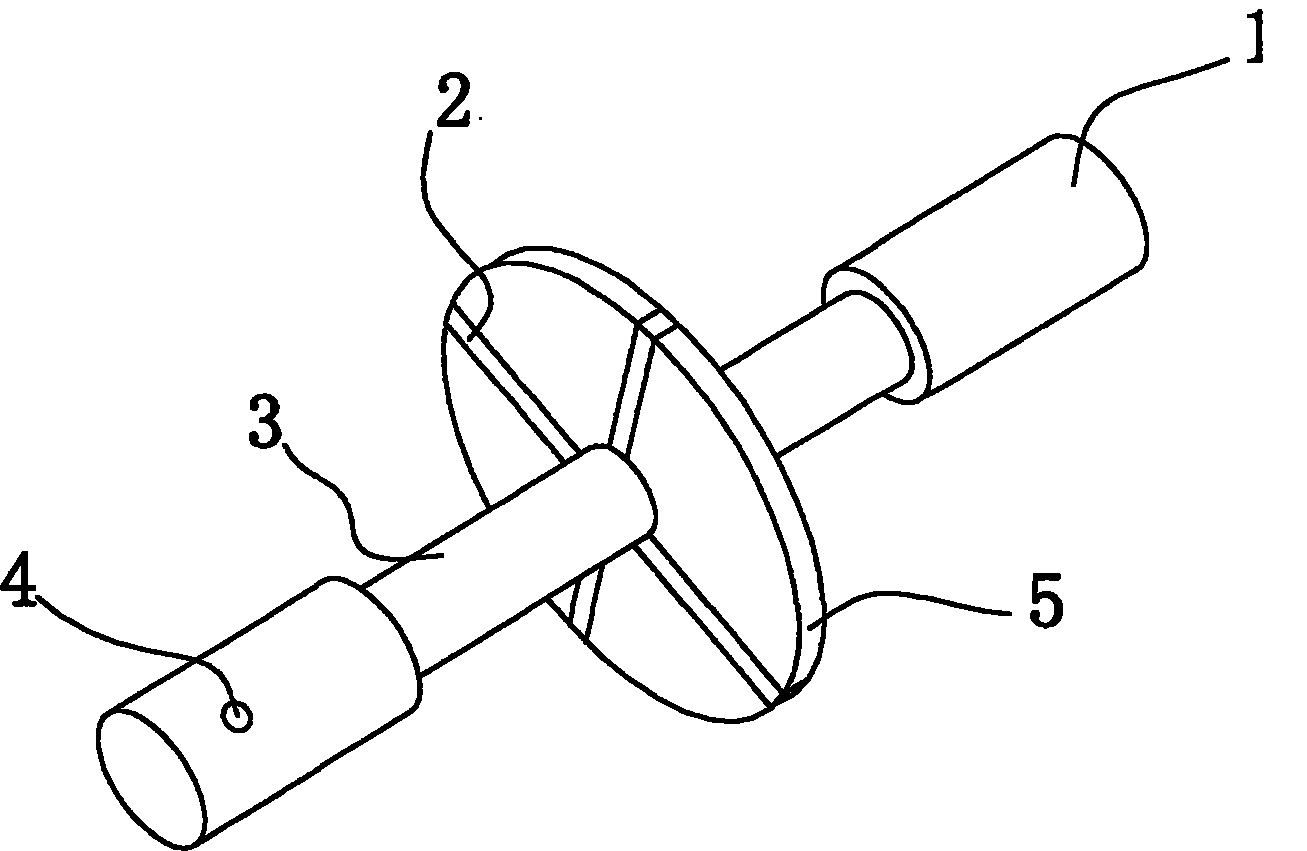


窗体底端
